# Simultaneous Quantification of Serum Nonesterified and Esterified Fatty Acids as Potential Biomarkers to Differentiate Benign Lung Diseases from Lung Cancer

**DOI:** 10.1038/srep34201

**Published:** 2016-09-30

**Authors:** Junling Ren, Dan Zhang, Yujie Liu, Ruiqing Zhang, Huiling Fang, Shuai Guo, Dan Zhou, Mo Zhang, Yupin Xu, Ling Qiu, Zhili Li

**Affiliations:** 1 Department of Biophysics and Structural Biology, Institute of Basic Medical Sciences, Chinese Academy of Medical Sciences & School of Basic Medicine, Peking Union Medical College, Beijing 100005, P. R. China; 2 Department of Clinical Laboratory, Peking Union Medical College Hospital, Chinese Academy of Medical Sciences & Peking Union Medical College, Beijing, 100730, P. R. China

## Abstract

In this study, we have employed graphene oxide as a matrix to simultaneously and directly quantify serum nonesterified and esterified fatty acids (FAs) using matrix-assisted laser/desorption ionization-Fourier transform ion cyclotron resonance mass spectrometry (MALDI-FTICR MS). Twelve serum nonesterified FAs combined with their individual esterified FAs (*i.e.*, C_16:0_, C_16:1_, C_18:0_, C_18:1_, C_18:2_, C_18:3_, C_20:2_, C_20:3_, C_20:4_, C_20:5_, C_22:5_, and C_22:6_) were quantified based on their calibration curves with the correlation coefficients of >0.99, along with the analytical time of <1 min each sample. As a result, serum levels of twelve total FAs (TFAs) in 1440 serum samples from 487 healthy controls (HCs), 479 patients with benign lung diseases (BLDs) and 474 patients with lung cancer (LC) were determined. Statistical analysis indicated that significantly increased levels of C_16:0_, C_16:1_, C_18:0_, C_18:1_, C_18:3_, C_20:3_, and C_22:6_ and decreased levels of C_20:5_ were observed in LC patients compared with BLDs. Receiver operating characteristic (ROC) analysis revealed that panel a (C_18:2_, C_20:3_, C_20:4_, C_20:5_, C_22:5_, and C_22:6_), panel b (C_18:0_, C_20:4_, C_20:5_, and C_22:6_), and panel c (C_16:1_, C_18:0_, C_18:1_, C_20:3_, and C_22:6_) have exhibited good diagnostic ability to differentiate BLDs from LC relative to clinical uses of tumor markers (CEA and Cyfra 21-1).

Lung cancer (LC) is the leading cause of cancer-related death worldwide for both men and women^1^, and a majority of LC patients present with inoperable at diagnosis and poor prognosis^2^. Despite the great progress made in recent years against LC, LC patients still have a low 5-year survival rate of approximately 18% in USA^3^. Currently, LC diagnosis and screening mainly depend on biopsy and high-resolution (or low-dose) computed tomography^4^,^5^. However, biopsy and computed tomography are not desirable to frequently detect tumors due to their invasive and high-cost. In addition, several circulating tumor markers, such as carcinoembryonic antigen (CEA) and cytokeratin 19 fragment antigen 21-1 (Cyfra 21-1), are the best known tumor markers for LC^6^, show poor sensitivities of 11~69% for LC detection^2^,^6^,^7^. Therefore, it is necessary to screen economic, simple, and noninvasive biomarkers for differentiating LC from benign lung diseases (BLDs).

Fatty acids (FAs) are the major structural components of lipids that involve in many biological functions, such as energy storage, signaling molecules^8^, and cellular messengers^9^. In addition, FAs have diverse structure and function in metabolism, cell, and tissue responses^10^,^11^. The sum of circulating free FAs (or nonesterified FAs) and esterified FAs from the circulating lipids may reflect the overall metabolism of endogenous and dietary FAs^12^,^13^. Previous studies have shown that serum total fatty acids (TFAs) including nonesterified FAs and esterified FAs not only generate great impact on human health but also involve in many biological functions, such as inflammatory response^14^,^15^. Emerging evidence indicates that free FAs are closely associated with insulin resistance and cancers^16^,^17^,^18^,^19^ and the esterified FAs play essential roles in regulating membrane fluidity, stability, and permeability, as well as in influencing the behaviors of membrane-bound enzymes and receptors^20^,^21^. The esterified FAs are associated with cancers^22^,^23^,^24^ and serum levels of TFAs are closely related with nutritional and metabolic disorders^13^,^25^.

Traditional analysis of circulating TFAs is usually performed using gas chromatography-mass spectrometry or liquid chromatography-mass spectrometry, both of which require complicated and time-consuming sample preparation^26^,^27^,^28^. Lipids analysis by matrix-assisted laser desorption/ionization -mass spectrometry (MALDI-MS) presents a lot of advantages, such as high detection speed, simplicity, and high salt tolerance. Recently, MALDI-MS has been applied to quantify metabolites *in vivo* without chromatographic separation^16^,^29^.

In this study, we have employed graphene oxide (GO) with efficient energy transfer, large thermal conductivity, and excellent electronic transportation^30^ as a matrix to ionize lipids in combination with collision induced dissociation to fragment phospholipids so that they become a novel powerful approach to simultaneously and directly quantify serum TFAs using MALDI-Fourier transform ion cyclotron resonance mass spectrometry (FTICR MS). Finally, twelve serum TFAs (*i.e.*, C_16:0_, C_16:1_, C_18:0_, C_18:1_, C_18:2_, C_18:3_, C_20:2_, C_20:3_, C_20:4_, C_20:5_, C_22:5_, and C_22:6_) were simultaneously quantified. Statistical analysis indicated that serum levels of TFAs are closely associated with physiopathological states and that the combination of C_18:2_, C_20:3_, C_20:4_, C_20:5_, C_22:5_, and C_22:6_, the combination of C_18:0_, C_20:4_, C_20:5_, and C_22:6_, and the combination of C_16:1_, C_18:0_, C_18:1_, C_20:3_, and C_22:6_ have shown good diagnostic capability to differentiate BLD patients from LC patients compared with CEA and Cyfra 21-1.

## Results

### Generating serum TFAs using optimal MS condition

**Figure 1:**
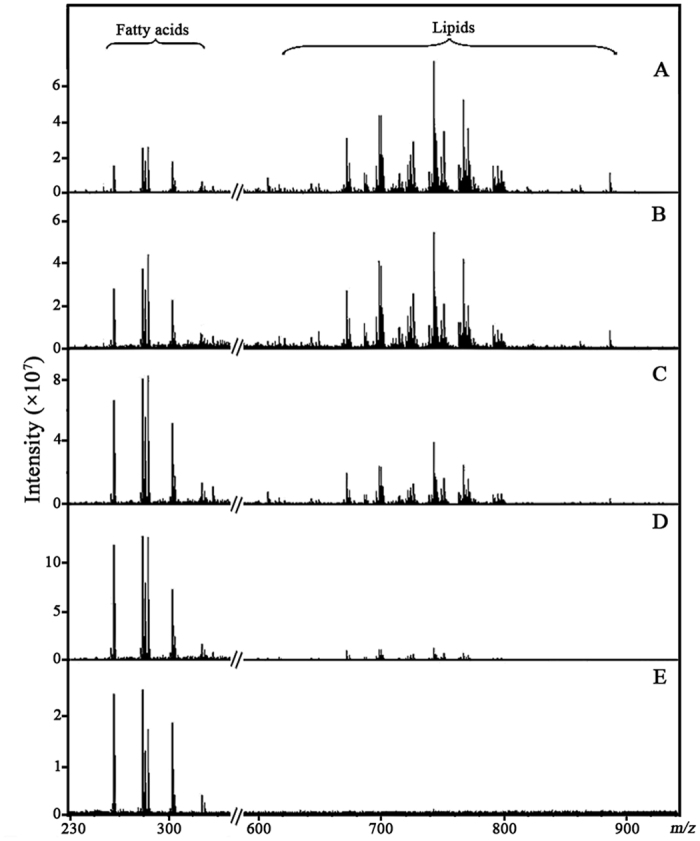
Effect of laser power and the skimmer 1 voltage on the intensity of serum TFAs using GO as a MALDI matrix. (**A**) laser power of 30%, skimmer 1 voltage of −10 V; (**B**) laser power of 50%, skimmer 1 voltage of −10 V; (**C**) laser power of 50%, skimmer 1 voltage of −45 V; (**D**) laser power of 90%, skimmer 1 voltage of −45 V; (**E**) laser power of 90%, skimmer 1 voltage of −100 V.

To establish an optimal MS condition for fragmenting phospholipids and generating serum TFAs using the QC sample as a model sample, we first optimized two important parameters: MALDI laser power and the skimmer 1 voltage of FTICR MS using GO as a MALDI matrix^31^. When the laser power was set to 30%, 50%, or 90% at both the skimmer 1 voltage of −10 V and−45 V, respectively, the intensities of different serum phospholipids were decreased gradually, while those of serum TFAs were increased (Fig. 1A–D). It should be noted that when the skimmer 1 voltage was set up to −100 V, the intensities of serum TFAs were dramatically decreased and phospholipids almost disappeared (Fig. 1E). Finally, it is found that most of the serum phospholipids were fragmented and abundant serum TFAs were generated with laser power of 90% and the skimmer 1 voltage of −45 V (Fig. 1D). As a result, the MS condition was optimal as laser power of 90% and the skimmer 1 voltage of −45 V.

**Figure 2:**
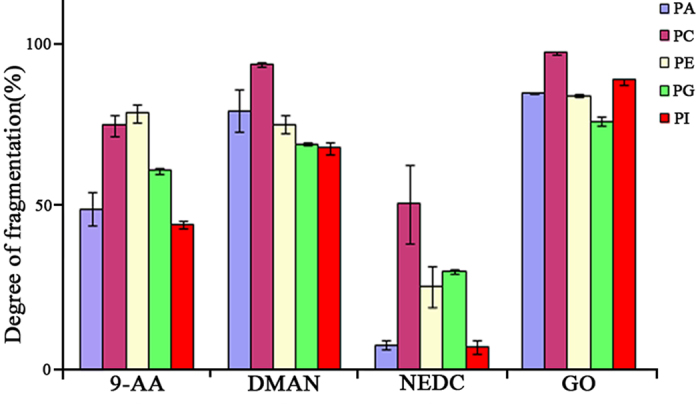
Effect of different MALDI matrixes on the fragmentation reaction of different types of phospholipids under the laser power of 90% and the skimmer 1 voltage of −45 V.

To assess the effect of different MALDI matrixes on the degree of the fragmentation of different phospholipids, several standard phospholipids (*i.e.*, PA(18:1/18:1), PE(18:1/18:1), PC(18:1/18:1), PI(16:0/18:2) and PG(16:0/16:0)) were selected as model compounds. As shown in Fig. 2, a comparison of commonly used matrixes (*i.e.*, 9-AA, DMAN, and NEDC) and GO shows that GO matrix has exhibited the best performance to fragment all of the above-mentioned standard phospholipids, along with the fragmentation degree of >80%, while NEDC matrix shows the lowest degree of fragmentation for all above-mentioned standard phospholipids compared with other matrixes. Therefore, GO was selected as the MALDI matrix to analyze serum extracts.

### Simultaneous quantification of serum TFAs

Based on the working standard solutions, the calibration curves of C_16:0_, C_16:1_, C_18:0_, C_18:1_, C_18:2_, C_18:3_, C_20:4_, and C_22:6_ were constructed with their correlation coefficients of >0.99 (Table 1), and their linearity ranges, LODs, and spike-and-recovery are also listed in Table 1. The reproducibility of the eight FAs is less than 18.0% (Table 1). Their intraday RSDs were from 6.2% to 10.7% and their interday RSDs were from 9.5% to 15.1% (Supplementary Table S1). The spike-and-recovery in triplicate at three different concentrations of all eight FAs were between 78.8% and 110.0%.

**Figure 3:**
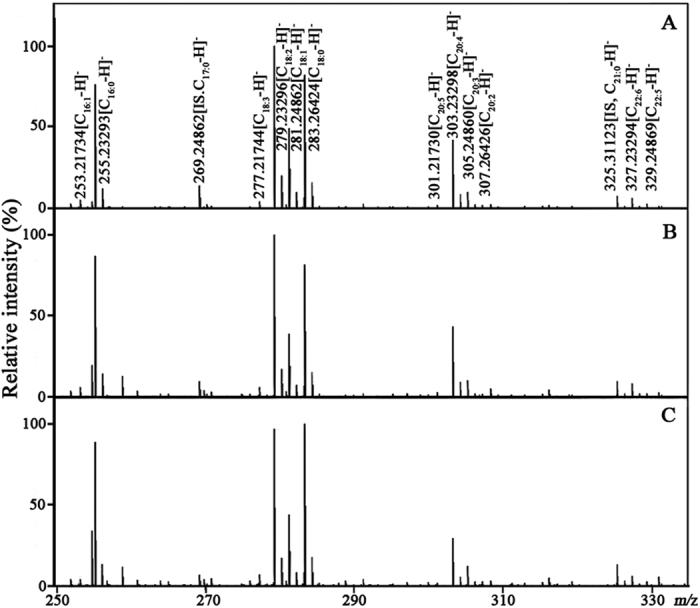
Representative mass spectra of serum TFAs from one HC (**A**), one BLD patient (**B**), and one LC patient (**C**) in negative ion mode.

**Figure 4:**
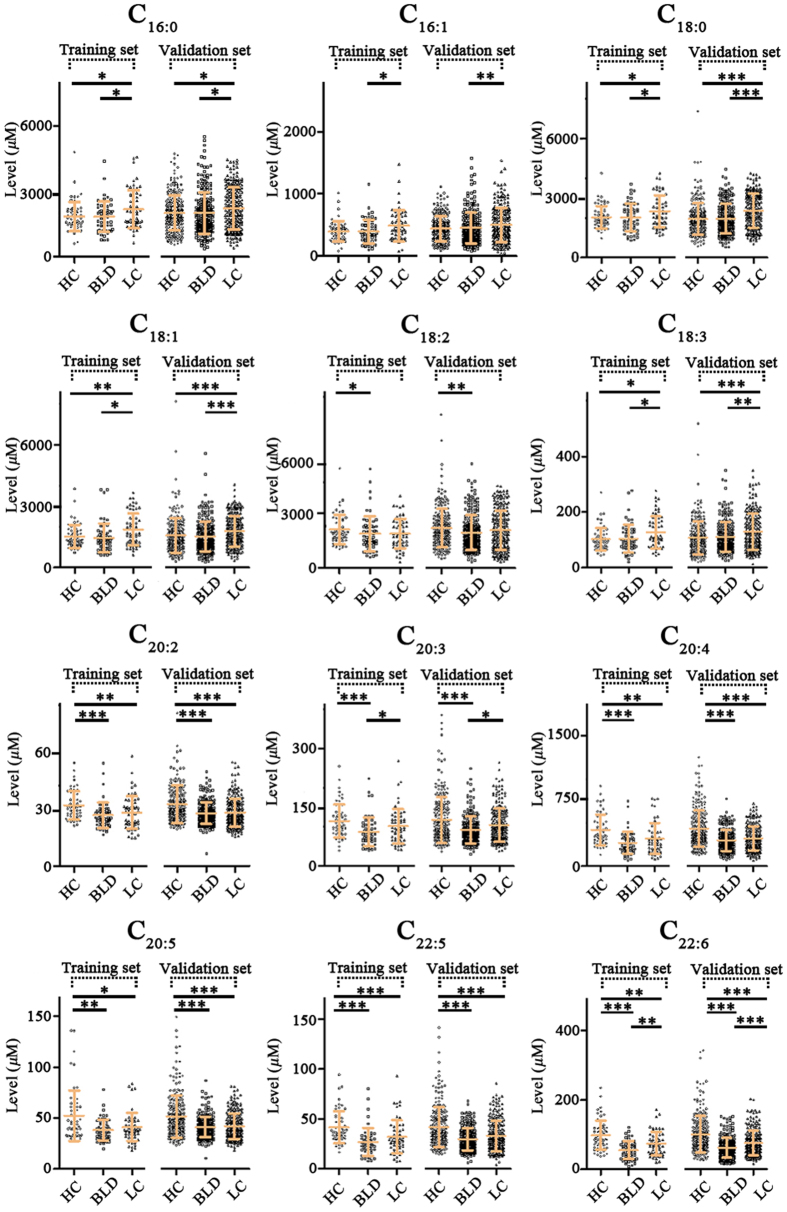
Scatter plots of the levels of serum TFAs in the training set and validation set. *p < 0.05; **p < 0.01; ***p < 0.001.

Representative mass spectra of serum TFAs from one HC, one BLD patient, and one LC patient are shown in Fig. 3. Serum TFAs quantified in this study were identified based on their observed accurate *m/z* values relative to the theoretical values with a mass error of <±0.00025 Da and reliable isotopic distributions with the RSDs of <2.0% between the observed and theoretical intensities (Supplementary Table S2). The levels of serum TFAs were calculated based on their respective calibration curves (Table 1) and the resulting data are shown in Fig. 4.

### Associations of the levels of serum TFAs with gender and age

Comparison of the levels of serum TFAs between female and male in each physiopathological state (For HCs, females: n = 212, age: 47.3 ± 10.3 years old; males: n = 275, age: 48.5 ± 10.1 years old. For BLD patients, females: n = 212, age: 55.7 ± 9.0 years old; males: n = 234, age: 55.2 ± 9.5 years old. For LC patients, females: n = 238, age: 57.5 ± 8.4 years old; males: n = 236, age: 57.9 ± 8.3 years old) and all participants (females: n = 745, age: 53.4 ± 10.2 years old; males: n = 695, age: 53.7 ± 10.2 years old) was performed using Mann-Whitney U test. The statistical analysis indicated that there is no statistical significance between the levels of serum TFAs and gender in each physiopathological state and between three different states (*p* > 0.05, Supplementary Table S3).

The effect of age on the levels of serum TFAs for HCs was also analyzed based on four different age groups (*i.e.*, group 1, 30~39 years old (n = 114); group 2, 40~49 years old (n = 157); group 3, 50~59 years old (n = 138), and group 4, 60~70 years old (n = 78)) using one-way ANOVA with LSD test after data were transformed to normal distribution. It was found that the levels of C_18:2_, C_20:2_, C_20:3_, C_20:4_, C_20:5_, C_22:5_, and C_22:6_ were significantly correlated with age (*p* < 0.05, Supplementary Table S4). However, there is no statistical difference except for C_18:3_ between group 3 and group 4 for BLD patients (*p* = 0.032, Supplementary Table S5) and LC patients (Supplementary Table S6).

### Association of changes in the levels of serum TFAs with physiopathological states

**Figure 5:**
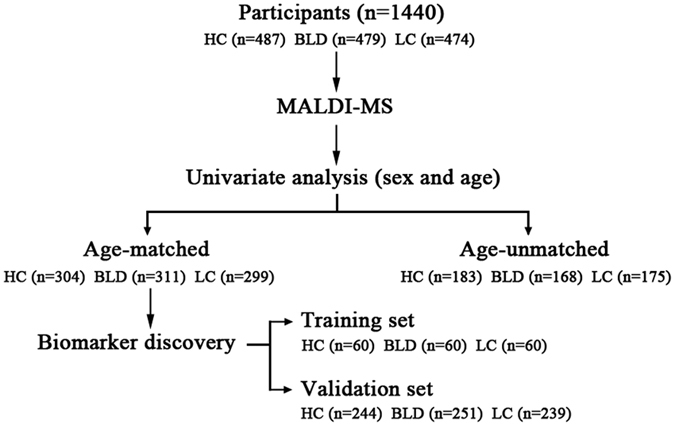
The flowchart of study design.

Based on the above-mentioned results, the 914 age-matched participants, including 304 HCs, 311 BLD patients, and 299 LC patients were selected to screen biomarkers for differentiating different physiopathological states (Table 2). In order to obtain high accurate diagnostic biomarkers, these participants were further classified randomly into a training set and a validation set (Fig. 5). In the training set study, as shown in Fig. 4, the levels of C_18:2_, C_20:2_, C_20:3_, C_20:4_, C_20:5_, C_22:5_, and C_22:6_ in BLD patients were significantly decreased relative to HCs. Significant increase in the levels of C_16:0_, C_18:0_, C_18:1_, and C_18:3_ and decrease in the levels of C_20:2_, C_20:4_, C_20:5_, C_22:5_, and C_22:6_ in LC patients were observed compared with HCs. However, remarkable increase in the levels of C_16:0_, C_16:1_, C_18:0_, C_18:1_, and C_18:3_ and decrease in the levels of C_20:3_ and C_22:6_ in LC patients were detected compared with BLD patients. In addition, an independent validation set also proved the above-mentioned change trends of serum TFAs in different physiological states (Fig. 4), and all *p* values are listed in Supplementary Table S7.

### Diagnostic ability of serum TFAs

The AUC values, sensitivities, specificities, and cut-off values of serum TFAs panels are listed in Table 3. For the training set, a combination of C_18:2_, C_20:3_, C_20:4_, C_20:5_, C_22:5_, and C_22:6_, namely panel a, has shown a powerful capability to differentiate HCs from BLD patients, with the AUC value of 0.863. A combination of C_18:0_, C_20:4_, C_20:5_, and C_22:6_, namely panel b, has a powerful ability to differentiate HCs from LC patients, with the AUC value of 0.729. A combination of C_16:1_, C_18:0_, C_18:1_, C_20:3_, and C_22:6_, namely panel c, is a good predictor for distinguishing BLD from LC patients, with the AUC value of 0.752. To validate the diagnostic ability of the above-mentioned panels, an independent validation study was performed. As shown in Table 3, the panels a, b, and c all have good capability to differentiate between HCs, BLDs, and LC based on the cut-off values obtained in the training set, with the AUC values of 0.781, 0.759, and 0.703, respectively. In addition, based on these cut-off values, each of three panels has shown a good capability to differentiate HC from BLDs plus LC, with the AUC values of >0.74 (Table 4), and it should be noted that the AUC values of three individual panels to distinguish HC plus BLDs from LC were still more than 0.64 (Table 4).

### Levels of serum tumor markers

In this study, serum tumor markers, CEA and Cyfra 21-1, were also measured in accordance with the manufacturer’s instructions and their median and ranges are listed Table 5. It is found that both CEA and Cyfra 21-1 were significantly increased in BLD or LC patients relative to HC (*p* < 0.001). However, no statistical differences in the level of serum Cyfra 21-1 was observed between BLD patients and LC patients.

### Comparison of diagnostic ability between serum tumor markers and serum TFA panels

Based on the cut-off values of serum CEA of 5.0 ng/mL, Cyfra 21-1 of 3.5 ng/mL and a combination of CEA and Cyfra 21-1 of 0.5, their AUC values were calculated to differentiate HC from BLDs, HC from LC or BLDs from LC. As shown in Table 6, CEA and Cyfra 21-1 present a similar AUC values as the serum TFA panels to differentiate HCs from BLDs patients or LC patients, along with high specificities and low sensitivities for CEA and Cyfra 21-1 compared with serum TFA panels. It is worth noting that serum TFA panels with the AUC values of 0.706~0.732 have shown high diagnostic accuracy to differentiate BLDs from LC relative to serum tumor markers with the AUC values of 0.521~0.588. In addition, ROC analysis indicated that the diagnostic ability of CEA, Cyfra 21-1 and a combination of CEA and Cyfra 21-1 with the AUC values of 0.739~0.829 is similar to that of serum TFA panels with the AUC values of 0.735~0.760 between HC and BLDs plus LC (Table 7), while serum TFA panels have exhibited a slightly better diagnostic ability to differentiate HC plus BLDs from LC compared with serum tumor markers (Table 7).

## Discussion

The detection of serum TFAs is usually performed using gas chromatography-mass spectrometry, along with a complicated and time-consuming sample preparation: lipid extraction, hydrolysis, and methylation^32^. In the present study, we developed a highly efficient method to generate esterified FAs and to detect directly and simultaneously serum nonesterified and esterified FAs through optimizing the skimmer 1 voltage of MS in combination with GO as a MALDI matrix. The calibration equations of C_16:0_, C_16:1_, C_18:0_, C_18:1_, C_18:2_, C_18:3_, C_20:4_, and C_22:6_ were constructed with the correlation coefficients of >0.99 based on their mixture standard working solutions. Their LODs were between 0.1 μM and 2.2 μM. 144 mass spectra of the QC sample were analyzed with a relative standard deviation of 18% for all analytes. The spike-and-recovery experiments on the basis of three different concentrations of FAs indicate that their recoveries were in the range from 78.8% to 110.0%. Our data indicate that the stability and precision of this method are acceptable for complex biological sample analysis. In term of the established method, twelve TFAs from 1440 serum samples were rapidly and simultaneously quantified and the measured concentrations of serum TFAs in the present study are similar to the previously published results obtained using gas chromatograph-mass spectrometry^13^,^33^.

Statistic analysis indicated that gender-specific difference was observed neither in the levels of twelve serum TFAs in each physiopathological state nor between three physiopathological states (Supplementary Table S3). It is worth noting that age-specific differences were only detected in the levels of some TFAs including C_18:2_, C_20:2_, C_20:3_, C_20:4_, C_20:5_, and C_22:6_ in HCs and C_18:3_ in BLD patients (Supplementary Tables S4 and S5), while for LC patients, no age-specific differences was observed, which are in line with previous reports^19^,^34^. Our findings indicate that different metabolic mechanisms between HC, BLDs, and LC might exist. Based on the age-matched samples (Table 2), statistic analysis indicated that the changes in the levels of serum TFAs between three different physiological states still present significantly statistical differences (Supplementary Table S7), further indicating the different metabolic mechanisms of serum TFAs between three physiopathological states.

Previous studies indicate that FA synthase has been found to be a hyperactivity in many cancers including LC^35^,^36^. Increase in the levels of C_16:0_, C_18:0_, and C_18:1_ in LC patients may be ascribed to the overexpression of FA synthase to sustain the increasing demand of energy during cancer cell proliferation^37^. It is found that α-linolenic acid (C_18:3_ n-3) can reduce the levels of reactive oxygen species produced by macrophages, resulting in the imbalance between oxidant and antioxidant systems in LC patients^38^. Decreased levels of C_20:2_, C_20:3_, C_20:4_, C_20:5_, C_22:5_, and C_22:6_ in both BLD and LC patients are in agreement with previous studies^26^,^27^,^39^, which may result in decreased membrane fluid to protect cancer cells from oxidative stress. In addition, C_20:2_, C_20:3_, C_20:4_, C_20:5_, C_22:5_, and C_22:6_ are the precursors of eicosanoid which are associated with inflammation, autoimmunity, and cancer^15^,^40^,^41^, and inflammation could influence tumor lipid metabolism^42^,^43^. Under inflammatory conditions, tumor cells may decrease the levels of endogenous inflammatory molecules to escape from immune attack and cell apoptosis^44^, which is in agreement with the decreased levels of C_20:2_, C_20:3_, C_20:4_, C_20:5_, C_22:5_, and C_22:6_ in both BLDs and LC.

These results further prove that lipid metabolism in HC and BLDs is significantly different from that in LC. The human body can produce all FAs except for C_18:2_ n-6 and C_18:3_ n-3^45^. C_18:2_ n-6, which affects gene expression^46^, is the precursor of n-6 series of FAs. Lipid mediators (e.g., prostaglandins, thromboxanes, and leukotrienes) play proinflammatory and angiogenic functions and involve in several pathologic progresses, which are generated primarily through oxidative pathways from C_20:4_^28^. In the present study, decreased C_18:2_ in BLDs and decreased C_20:4_ in both BLDs and LC might be associated with changes in inflammatory response. In addition, the combinations of serum TFAs have exhibited powerful diagnostic ability to differentiate BLDs from LC with the AUC values of 0.706~0.732 relative to serum tumor markers with the AUC values of 0.521~0.588 (Tables 6 and 7), indicating that lipid mechanisms are closely correlated with different physiological states.

Our study has some limitations. First, due to the lack of the detailed clinical information on LC staging, early stage diagnostic ability of serum TFAs could not be preformed. Second, the location of the double bonds of each unsaturated FAs has not been designed due to no gas chromatography separation and corresponding standard compounds. Finally, non complete fragmentation of serum phospholipids may affect the diagnostic capability of serum TFAs.

## Conclusions

In the present study, based on the optimized parameters of MS in combination with the physicochemical properties of GO as a MALDI matrix, we developed a rapid and simultaneous quantification method of 12 serum FAs including nonesterified and esterified FAs via the direct fragmentation of phospholipids using MALDI-MS without hydrolysis and methylation of FAs. The correlation coefficients of the calibration curves were large than 0.99, with a linear dynamic range of 3 orders of magnitude and the LODs of 0.1~2.2 μM. The levels of serum TFAs in 1440 serum samples from three different physiopathological states reveal that lipid metabolic mechanisms are closely correlated with the physiopathological states. ROC analysis indicated that three different TFA panels have exhibited good diagnostic capability to differentiate among three different physiopathological states with the AUC values of >0.7 compared with serum tumor markers. Especially for differentiating BLDs from LC, the AUC values of serum TFA panels are 0.706~0.732, while those of serum tumor markers are 0.521~0.588. Taken together, our findings indicate that lipid metabolism is deeply involved in changes in physiological state, and our data may offer a stepping stone of new biomarker panels for differentiating BLDs from LC.

## Methods

### Chemicals and reagents

Pentadecanoic acid (C_15:0_), palmitic acid (C_16:0_), palmitoleic acid (C_16:1_), heptadecanoic acid (C_17:0_), stearic acid (C_18:0_), oleic acid (C_18:1_), linoleicacid (C_18:2_), linolenic acid (C_18:3_), nonadecanoic acid (C_19:0_), arachidonic acid (C_20:4_), heneicosanoic acid (C_21:0_), docosahexaenoic acid (C_22:6_), L-α-phosphatidylinositol sodium salt (PI, PI(16:0/18:2)), 9-aminoacridine (9-AA), 1,8-bis(dimethylamino)naphthalene (DMAN), and N-(1-naphthyl) ethylenediamine dihydrochloride (NEDC) were purchased from Sigma-Aldrich Chemicals (St. Louis, MO, USA). 1,2-di-(9Z-octadecenoyl)-sn-glycero-3-phosphate (sodium salt) (PA(18:1(9Z)/18:1(9Z))), 1,2-di-(9E-octadecenoyl)-sn-glycero-3-phosphocholine (PC(18:1(9E)/18:1(9E))), 1,2-di-(9E-octadecenoyl)-sn-glycero-3-phosphoethanolamine (PE(18:1(9E)/18:1(9E))) and 1,2-dipalmitoyl-sn-glycero-3-phospho-(1′-rac-glycerol) (sodium salt) (PG(16:0/16:0)) were purchased from Avanti Polar lipids (Alabaster, AL, USA). GO was from Nanjing JCNANO Technology Co., Ltd (Nanjing, China). HPLC-grade isopropanol and hexane were from Fisher Scientific (Pittsburg, PA, USA). Ultrapure water was purified by a Milli-Q system (Millipore, MA, USA).

### Participants and study design

In this study, a total of 1440 overnight (more than 10 hours) fasting serum samples were collected in Peking Union Medical College Hospital (Beijing, China). Serum samples are the remaining sera after routine physical examination or clinical examination. All samples were stored under −80 °C until use. Healthy controls (HCs) without any aberrant clinical appearance and pathomorphology were included. BLDs were diagnosed based on the clinical diagnostic criterion and LC was further confirmed by cytological or histological examination of tumor tissue. Study design is shown in Fig. 5. Age-unmatched participants (*i.e.*, 183 HCs, 168 BLD patients, and 175 LC patients) were excluded, and the clinical characteristics of the age-matched participants are shown in Table 2. Informed consent were obtained from all participants and the study was approved by the Ethics Review Board at the Institute of Basic Medical Sciences, Chinese Academy of Medical Sciences. All experiments were performed in accordance with relevant guidelines and regulations.

### The levels of CEA and Cyfra 21-1

Serum tumor marker levels were determined with a CEA and Cyfra 21-1 test kit (Roche Diagnostic GmbH, Mannheim, Germany) using a Cobas e601 analyzer. The cut-off values were 5.0 ng/mL for CEA and 3.5 ng/mL for Cyfra 21-1 as recommended by the manufacture’s instruction.

### Preparation of stock standard solutions

C_17:0_ and C_21:0_ were selected as internal standards (ISs), and their mixed stock solution was prepared in isopropanol at the concentrations of 375 μM C_17:0_ and 75 μM C_21:0_. The standard solutions of 6.60 mM C_16:0_, 1.65 mM C_16:1_, 10.00 mM C_18:0_, 10.00 mM C_18:1_, 10.00 mM C_18:2_, 0.826 mM C_18:3_, 3.30 mM C_20:4_, and 1.65 mM C_22:6_ were prepared in isopropanol, respectively. Then equal volume of the above-mentioned standard solutions was pooled into the mixed standard solution followed by 1-fold dilution as the stock standard solution at the final concentrations of 412.5 μM C_16:0_, 103.1 μM C_16:1_, 625.0 μM C_18:0_, 625.0 μM C_18:1_, 625.0 μM C_18:2_, 51.6 μM C_18:3_, 206.3 μM C_20:4_, and 103.1 μM C_22:6_.

### Sample preparation

Lipids were extracted from serum sample on the basis of the approach used by Hara *et al*.^47^, with a slight modification. Briefly, after thawing at 4 °C, 10 μL of serum sample was transferred into a 1.5 mL eppendorf tube containing 10 μL ISs (37.5 μM C_17:0_ and 7.5 μM C_21:0_), followed by the addition of 135 μL of hexane/isopropanol (2:1, v:v) and 45 μL ultrapure water. The resulting mixture was vortexed for 1 min and then stored at −20 °C overnight. After the mixture was centrifuged at 19,000 × g for 30 min at 4 °C, 20 μL of the supernatant was transferred into a new tube and air-dried. The dried sample was stored at −80 °C until use. 10 μL of isopropanol/water (1:1, v:v) was added to re-dissolve the air-dried samples for mass spectrometric analysis.

### Mass spectrometry analysis

The GO solution (0.5 mg/mL in water) was sonicated for 2 h, followed by the centrifugation at 13,000 × *g* to remove the unexfoliated GO particles. Then the supernatant was collected for further use as a MALDI matrix. 0.3 μL of the GO solution was first pipetted on the MTP AnchorChip^TM^ plate (Bruker Daltonics, Billerica, MA, USA) and air-dried prior to the addition of 0.3 μL of the redissolved sample onto the GO matrix for mass spectrometric analysis.

All experiments were performed using a 9.4 T Apex-ultra^TM^ hybrid Qh-FTICR MS (Bruker Daltonics, Billerica, MA, USA) equipped with a 355 nm Nd:YAG Smartbeam II 200 Hz laser in negative ion mode. Instrument calibration was performed using a mixture of C_15:0_ at *m/z* 241.21730, C_17:0_ at *m/z* 269.24860, C_19:0_ at *m/z* 297.27990, and C_21:0_ at *m/z* 325.31120 in negative ion mode. Mass spectrum of each sample was acquired over the m/z range of 150~400 with the resolution of 200,000 at m/z 400, along with 100 laser shots per scan and the skimmer 1 voltage of −45 V in negative ion mode. The fragmentation degree of the model compounds was calculated based on the equation (1).


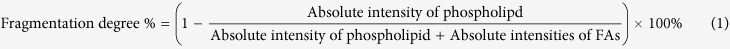


### Statistical analysis

Mass spectral data were obtained using ApexControl 3.0.0 (Bruker Daltonics). After isotopic deconvolution, the resulting data were transferred to Microsoft Excel, and the half of baseline strength in each spectrum was adopted as their intensities of missing serum TFAs. Univariate analysis was performed using non-parametric Mann-Whitney U test. One-way analysis of variance (ANOVA) with Fisher’s least significant test was used to evaluate the effect of age on the levels of serum TFAs. Non-normally distributed data were transformed into normal distribution before statistical analysis. Receiver operating characteristics (ROC) curve analysis was used to calculate the area under the ROC curve (AUC), cut-off values, sensitivities, and specificities. The prediction model was further confirmed by an independent validation set based on the cut-off values obtained in the training set. Statistical analyses were performed using SPSS software (version 16.0, Chicago, IL, USA). In all cases, a p value less than 0.05 was considered to be statistically significant.

### Identification of serum TFAs

Serum TFAs were identified as previously described^19^. Briefly, serum TFAs were identified based on their observed accurate m/z values relative to theoretical values of <±0.00025 Da and their observed distributions of isotopic abundance relative to theoretical distributions of <2.0%.

### Method validation for quantitative analysis

The reliability of MALDI-MS for quantitative analysis of FAs was validated based on the linearity, limit of detection (LOD), stability, precision, and spike-and-recovery experiments.

To construct the calibration curves of each FA, the mixed stock standard solution was diluted to four different concentrations (*i.e.*, 3.3, 6.6, 16.5, and 82.5-fold), respectively, and finally, five working standard solutions were obtained. The calibration curves between the intensity ratios of individual FAs to ISs (the final concentrations of 37.5 μM C_17:0_ and 7.5 μM C_21:0_) versus their corresponding concentration ratios were constructed based on the above-mentioned working standard solutions. C_17:0_ as an IS was for quantifying the levels of C_16:0_, C_16:1_, C_18:0_, C_18:1_, C_18:2_, and C_18:3_, and C_21:0_ as an IS was for quantifying the levels of C_20:2_, C_20:3_, C_20:4_, C_20:5_, C_22:5_, and C_22:6_. In addition, the calibration curve of C_20:4_ was also used for quantifying C_20:2_, C_20:3_, and C_20:5_ and the calibration curve of C_22:6_ was for quantifying C_22:5_ because their commercial standards are not available. Each of the working solution was analyzed three times and the results were shown as mean ± standard deviation. The LOD is defined as the concentration of each analytes at the signal-to-noise ratio of 3.

A pooled quality control (QC) serum sample obtained from the mixture of 5 HCs and 5 LC patients sera was analyzed once every 10 test samples. Finally, a total of 144 spectra of the QC sample were obtained in this study. The experimental stability and reproducibility were evaluated using the relative standard deviation (RSD) obtained based on the intensity ratios of the detected TFAs relative to their corresponding ISs. The experimental precision was assessed based on the intraday precision of three measured values of the QC sample on the same day and the interday precision of three measured values of the QC sample on three consecutive days.

To assess the extraction efficiency of serum phospholipids and FAs, the spike-and-recovery experiment was employed. Briefly, 10 μL of the QC serum sample in triplicate was mixed with 10 μL ISs, 127.5 μL hexane/isopropanol (2:1, v:v), and 42.5 μL water, and then three resulting solutions were spiked with 10 μL of three different concentrations of standard FAs, respectively. Finally, three different concentration solutions of the spiked FAs are listed as below. R1, the concentrations of the spiked FAs: 10.0 μM C_16:0_, 2.5 μM C_16:1_, 15.0 μM C_18:0_, 15.0 μM C_18:1_, 15.0 μM C_18:2_, 1.2 μM C_18:3_, 5.0 μM C_20:4_, and 2.6 μM C_22:6_; R2, the concentrations of the spiked FAs: 100.0 μM C_16:0_, 25.0 μM C_16:1_, 150.0 μM C_18:0_, 150.0 μM C_18:1_, 150.0 μM C_18:2_, 12.0 μM C_18:3_, 50.0 μM C_20:4_, and 26.0 μM C_22:6_; R3, the concentration of the spiked FAs: 150.0 μM C_16:0_, 37.5 μM C_16:1_, 225.0 μM C_18:0_, 225.0 μM C_18:1_, 225.0 μM C_18:2_, 18.0 μM C_18:3_, 75.0 μM C_20:4_, and 39.0 μM C_22:6._ Lipid extraction of these three solutions were performed as described at the section of sample preparation and each was analyzed in triplicate.

## Additional Information

**How to cite this article**: Ren, J. *et al*. Simultaneous Quantification of Serum Nonesterified and Esterified Fatty Acids as Potential Biomarkers to differentiate benign lung diseases from lung cancer. *Sci. Rep.* **6**, 34201; doi: 10.1038/srep34201 (2016).

## Supplementary Material

Supplementary Information

## Figures and Tables

**Table 1:** The linearity range, calibration curve, correlation coefficient (R^2^), limit of detection (LOD), and recovery of FAs.

FAs	Linearity (*n* = 3)			LOD (μM)	QC (%)	Recovery (%, *n* = 3)		
	FA (*μ*M)	Calibration curve	R^2^			R1	R2	R3
C_16:0_	5.0~412.5	Y = 1.184(±0.062)X + 0.256(±0.067)	0.996	0.5	<16	78.8	94.7	95.7
C_16:1_	1.3~103.1	Y = 0.427(±0.234)X + 0.054(±0.018)	0.998	0.1	<17	95.5	110.0	99.6
C_18:0_	7.5~625.0	Y = 1.176(±0.061)X−0.147(±0.204)	0.991	2.2	<17	92.8	79.6	93.9
C_18:1_	7.5~625.0	Y = 0.877(±0.081)X + 0.177(±0.236)	0.996	1.5	<17	92.7	95.2	97.9
C_18:2_	7.5~625.0	Y = 1.059(±0.041)X−0.139(±0.083)	0.996	1.9	<18	93.4	92.2	97.7
C_18:3_	0.6~51.6	Y = 1.405(±0.114)X + 0.011(±0.024)	0.995	0.3	<18	107.8	105.5	91.1
C_20:4_	2.5~206.3	Y = 1.047(±0.077)X−0.205(±0.269)	0.998	0.1	<18	96.6	86.1	91.9
C_22:6_	1.3~103.1	Y = 0.925(±0.087)X−0.031(±0.122)	0.993	0.3	<16	85.9	96.8	90.6

X: concentration ratios of individual FAs to ISs (37.5 μM for C_17:0_ and 7.5 μM for C_21:0_); Y: respective corresponding intensity ratios of FAs to ISs. R1: a mixture of 10.0 μM C_16:0_, 2.5 μM C_16:1_, 15.0 μM C_18:0_, 15.0 μM C_18:1_, 15.0 μM C_18:2_, 1.2 μM C_18:3_, 5.0 μM C_20:4_, and 2.6 μM C_22:6_; R2: a mixture of 100.0 μM C_16:0_, 25.0 μM C_16:1_, 150.0 μM C_18:0_, 150.0 μM C_18:1_, 150.0 μM C_18:2_, 12.0 μM C_18:3_, 50.0 μM C_20:4_, and 26.0 μM C_22:6_; R3: a mixture of 150.0 μM C_16:0_, 37.5 μM C_16:1_, 225.0 μM C_18:0_, 225.0 μM C_18:1_, 225.0 μM C_18:2_, 18.0 μM C_18:3_, 75.0 μM C_20:4_, and 39.0 μM C_22:6_.

**Table 2:** Characteristics of the participants.

	Training set			Validation set		
	HC (n = 60)	BLDs (n = 60)	LC (n = 60)	HC (n = 244)	BLDs (n = 251)	LC (n = 239)
**Sex (M/F)**	31/29	30/30	31/29	146/98	126/125	122/117
**Age (years)**						
Mean ± SD	54.4 ± 7.7	55.3 ± 7.2	54.1 ± 7.6	52.9 ± 8.9	52.8 ± 9.2	54.5 ± 8.7
Range	30~70	30~70	30~70	30~70	30~70	30~70
**Serum markers (M/F)**						
CEA				93/54	79/74	58/50
Cyfra 21-1				93/54	79/74	58/50
**Characteristics of BLDs (M/F)**						
Pulmonary infection		7/3			35/15	
Pulmonary shadow		3/0			13/19	
Pulmonary sarcoidosis		11/18			41/52	
Interstitial lung disease		2/1			8/11	
Pneumonia		2/2			3/8	
**Histology (M/F)**						
Non-small cell lung cancer			18/13			68/51
Small cell lung cancer			7/4			13/17
**Stage (M/F)**						
I and II			1/1			1/2
III and IV			10/6			34/19

HC: healthy control, BLDs: benign lung diseases, LC: lung cancer, M: male, F: female, SD: standard deviation.

**Table 3:** The AUC values, sensitivities, specificities, and cut-off values of different panels of serum TFAs.

	Panel a				Panel b				Panel c			
	AUC (95% CI)	Sens (%)	Spec (%)	Cut-off	AUC (95% CI)	Sens (%)	Spec (%)	Cut-off	AUC (95% CI)	Sens (%)	Spec (%)	Cut-off
**Training set**												
HC *vs.* BLDs	0.863(0.799~0.927)	68.3	81.7	0.6								
HC *vs.* LC					0.729(0.640~0.817)	68.3	65.0	0.5				
BLDs *vs.* LC									0.752(0.664~0.839)	70.0	73.3	0.5
**Validation set**												
HC *vs.* BLDs	0.781(0.741~0.821)	65.3	77.5	0.6								
HC *vs.* LC					0.759(0.717~0.801)	72.4	67.6	0.5				
BLDs *vs.* LC									0.703(0.657~0.749)	60.7	73.4	0.5

Panel a: C_18:2_, C_20:3_, C_20:4_, C_20:5_, C_22:5_, and C_22:6_; panel b: C_18:0_, C_20:4_, C_20:5_, and C_22:6_; panel c: C_16:1_, C_18:0_, C_18:1_, C_20:3_, and C_22:6_.

**Table 4:** The AUC values, sensitivities, specificities, and cut-off values of different panels of serum TFAs.

Panels	HC *vs.* BLDs plus LC			Cut-off	HC plus BLDs *vs.* LC		
	AUC (95% CI)	Sens (%)	Spec (%)		AUC (95% CI)	Sens (%)	Spec (%)
Panel a	0.742(0.705~0.779)	61.2	75.0	0.6	0.654(0.612~0.696)	66.9	55.6
Panel b	0.765(0.732~0.798)	70.5	70.1	0.5	0.682(0.645~0.718)	68.6	56.3
Panel c	0.746(0.709~0.783)	76.9	63.1	0.5	0.641(0.596~0.686)	73.2	53.5

Panel a: C_18:2_, C_20:3_, C_20:4_, C_20:5_, C_22:5_, and C_22:6_; panel b: C_18:0_, C_20:4_, C_20:5_, and C_22:6_; panel c: C_16:1_, C_18:0_, C_18:1_, C_20:3_, and C_22:6_.

**Table 5:** Serum levels of CEA and Cyfra 21-1 in HC, BLDs, and LC.

Serum markers	HC	BLDs	LC
CEA (ng/mL, median, range)	2.0(0.1~22.3)	4.0(0.2–1090.0)^a^	6.8(0.4~200.4)a, b
Cyfra 21-1 (ng/mL, median, range)	1.3(0.4~7.1)	2.0(0.5–172.9)^a^	1.9(0.8~36.9)

CEA, carcinoembryonic antigen; Cyfra 21-1, cytokeratin 19 fragments. ^a^HC *vs.* BLDs or LC, *p* < 0.001; ^b^BLDs *vs.* LC, *p* < 0.001.

**Table 6:** The AUC values, sensitivities, specificities, and cut-off values of serum tumor markers and TFA panels.

	HC *vs.* BLDs			HC *vs.* LC			BLDs *vs.* LC			Cut-off
	AUC (95% CI)	Sens (%)	Spec (%)	AUC (95% CI)	Sens (%)	Spec (%)	AUC (95% CI)	Sens (%)	Spec (%)	
**Serum markers**										
CEA	0.680(0.621~0.739)	42.2	92.5	0.781(0.718~0.844)	55.6	92.5	0.588(0.529~0.647)	56.0	57.5	5.0
Cyfra 21-1	0.753(0.701~0.806)	24.4	97.3	0.726(0.664~0.787)	19.4	97.3	0.549(0.489~0.609)	80.7	25.0	3.5
CEA plus Cyfra 21-1	0.813(0.765~0.861)	69.2	87.0	0.844(0.794~0.894)	62.0	91.8	0.521(0.460~0.581)	99.1	4.6	0.5
**Panels**										
Panel a	0.761(0.709~0.813)	60.7	80.3	0.748(0.689~0.806)	75.9	63.1	0.732(0.680~0.783)	60.2	78.4	0.6
Panel b	0.779(0.728~0.830)	79.2	63.3	0.769(0.712~0.826)	62.0	76.5	0.709(0.656~0.762)	63.7	69.9	0.5
Panel c	0.779(0.728~0.830)	86.7	59.9	0.744(0.684~0.803)	68.5	69.1	0.706(0.653~0.758)	65.9	70.6	0.5

Panel a: C_18:2_, C_20:3_, C_20:4_, C_20:5_, C_22:5_, and C_22:6_; panel b: C_18:0_, C_20:4_, C_20:5_, and C_22:6_; panel c: C_16:1_, C_18:0_, C_18:1_, C_20:3_, and C_22:6_.

**Table 7:** The AUC values, sensitivities, specificities, and cut-off values of serum tumor markers and TFA panels.

	HC *vs.* BLDs plus LC			Cut-off	HC plus BLDs *vs.* LC		
	AUC (95% CI)	Sens (%)	Spec (%)		AUC (95% CI)	Sens (%)	Spec (%)
**Serum markers**							
CEA	0.739(0.698~0.780)	50.5	92.5	5.0	0.673(0.612~0.733)	55.6	73.7
Cyfra 21-1	0.763(0.690~0.781)	21.6	97.3	3.5	0.580(0.520~0.639)	19.4	85.6
CEA plus Cyfra 21-1	0.829(0.794~0.864)	84.6	51.4	0.5	0.603(0.536~0.670)	41.7	80.8
**Panels**							
Panel a	0.735(0.688~0.782)	66.8	70.5	0.6	0.695(0.638~0.752)	60.2	75.0
Panel b	0.760(0.713~0.806)	76.8	62.4	0.5	0.701(0.644~0.751)	71.3	60.6
Panel c	0.741(0.692~0.789)	69.9	68.5	0.5	0.675(0.615~0.735)	60.2	67.2

Panel a: C_18:2_, C_20:3_, C_20:4_, C_20:5_, C_22:5_, and C_22:6_; panel b: C_18:0_, C_20:4_, C_20:5_, and C_22:6_; panel c: C_16:1_, C_18:0_, C_18:1_, C_20:3_, and C_22:6_.
