# Supplementary material for: Simultaneous Quantification of Serum Nonesterified and Esterified Fatty Acids as Potential Biomarkers to Differentiate Benign Lung Diseases from Lung Cancer
Source: Sci Rep. 2016 Sep 30;6:34201. doi: 10.1038/srep34201 (PMC5043242; doi:10.1038/srep34201)
Supplement: Supplementary Information [file srep34201-s1.pdf]

Supplementary Information for:

Simultaneous Quantification of Serum Nonesterified and Esterified Fatty Acids as Potential Biomarkers to Differentiate Benign Lung Diseases from Lung Cancer

Junling Ren[1], Dan Zhang[1], Yujie Liu[1], Ruiqing Zhang[1], Huiling Fang[2], Shuai Guo[1], Dan Zhou[1], Mo Zhang[1], Yupin Xu[1], Ling Qiu[2]*, Zhili Li[1]*

[1]Department of Biophysics and Structural Biology, Institute of Basic Medical Sciences, Chinese Academy of Medical Sciences & School of Basic Medicine, Peking Union Medical College, Beijing 100005, PR China

[2]Department of Clinical Laboratory, Peking Union Medical College Hospital, Chinese Academy of Medical Sciences & Peking Union Medical College,
Beijing, 100730, PR China.

***Corresponding author: Zhili Li**, Department of Biophysics and Structural Biology, Institute of Basic Medical Sciences, Chinese Academy of Medical Sciences & School of Basic Medicine, Peking Union Medical College, 5 Dongdan San Tiao, Beijing 100005, PR China. E-mail: lizhili@ibms.pumc.edu.cn,Tel/Fax: +86-10-69156479.

Ling Qiu, Department of Clinical Laboratory, Peking Union Medical College Hospital, Chinese Academy of Medical Sciences & Peking Union Medical College, Beijing, 100730, PR China. E-mail: lingqiubj@aliyun.com, Tel:+86-10-69159712

Table S1. Intraday and interday precision of the method

Precision (RSD%)	TFAs											
	$C_{16:1}$	$C_{16:0}$	$C_{18:3}$	$C_{18:2}$	$C_{18:1}$	$C_{18:0}$	$C_{20:5}$	$C_{20:4}$	$C_{20:3}$	$C_{20:2}$	$C_{22:6}$	$C_{22:5}$
Intraday (n = 3)	10.5	8.6	8.0	8.4	6.4	10.7	10.7	6.2	6.4	8.2	9.3	9.3
Interday (n = 3)	12.3	15.0	15.1	9.8	9.5	10.9	13.4	13.6	14.7	13.4	13.1	13.1

RSD: relative standard deviation.

Table S2. The accurate molecular weight and isotopic abundance of the detected TFAs

TFAs	Theoretical value		Experimental value		Mass error (Da)
	m/z	Iso. Abu.(%)	*m/z*	Iso. Abu.(%)	
$C_{16:0}$	255.23295[M-H]$^-$	100.000	255.23293[M-H]$^-$	100.000	-0.00002
	256.23637	17.738	256.23624	17.580	-0.00013
$C_{16:1}$	253.21730[M-H]$^-$	100.000	253.21734[M-H]$^-$	100.000	0.00004
	254.22072	17.715	254.22063	16.730	-0.00009
$C_{18:0}$	283.26425[M-H]$^-$	100.000	283.26424[M-H]$^-$	100.000	-0.00001
	284.26767	19.947	284.26763	19.415	-0.00004
$C_{18:1}$	281.24860[M-H]$^-$	100.000	281.24862[M-H]$^-$	100.000	0.00002
	282.25202	19.924	282.25202	18.839	0.00000
$C_{18:2}$	279.23295[M-H]$^-$	100.000	279.23296[M-H]$^-$	100.000	0.00001
	280.23636	19.901	280.23634	18.435	-0.00002
$C_{18:3}$	277.21730[M-H]$^-$	100.000	277.21744[M-H]$^-$	100.000	0.00014
	278.22071	19.878	278.22074	18.514	0.00003
$C_{20:2}$	307.26425[M-H]$^-$	100.000	307.26426[M-H]$^-$	100.000	0.00001
	308.26776	22.110	308.26752	21.088	0.00024
$C_{20:3}$	305.24860[M-H]$^-$	100.000	305.24860[M-H]$^-$	100.000	0.00000
	306.25201	22.087	306.25194	21.454	-0.00007
$C_{20:4}$	303.23295[M-H]$^-$	100.000.	303.23298[M-H]$^-$	100.000	0.00003
	304.23636	22.064	304.23633	20.672	-0.00003
$C_{20:5}$	301.21730[M-H]$^-$	100.000	301.21730[M-H]$^-$	100.000	0.00000
	302.22071	22.041	302.22077	23.204	0.00006
$C_{22:5}$	329.24860[M-H]$^-$	100.000	329.24869[M-H]$^-$	100.000	0.00009
	330.25201	24.250	330.25190	26.021	-0.00011
$C_{22:6}$	327.23295[M-H]$^-$	100.000	327.23294[M-H]$^-$	100.000	-0.00001
	328.23635	24.227	328.23630	23.220	-0.00005

Table S3. Comparison of serum TFAs levels between female and male in 1440 participants and each pathophysiological state using Mann-Whitney U test

TFAs	Comparison of all participants (p values)	Comparison within groups (p values)		
		HC	BLD	LC
$C_{16:0}$	0.825	0.588	0.937	0.051
$C_{16:1}$	0.651	0.699	0.960	0.528
$C_{18:0}$	0.576	0.949	0.385	0.813
$C_{18:1}$	0.150	0.191	0.589	0.950
$C_{18:2}$	0.500	0.396	0.953	0.548
$C_{18:3}$	0.276	0.408	0.717	0.538
$C_{20:2}$	0.634	0.551	0.359	0.986
$C_{20:3}$	0.442	0.212	0.887	0.714
$C_{20:4}$	0.243	0.284	0.894	0.498
$C_{20:5}$	0.837	0.129	0.415	0.089
$C_{22:5}$	0.672	0.454	0.812	0.236
$C_{22:6}$	0.367	0.572	0.209	0.093

Females: n = 745, age: 53.4 ± 10.2; males: n = 695, age: 53.7 ± 10.2. For HC group, females: n = 212, age: 47.3 ± 10.3; males: n = 275, age: 48.5 ± 10.1. For BLD group, females: n = 212, age: 55.7 ± 9.0; males: n = 234, age: 55.2 ± 9.5. For LC group, females: n = 238, age: 57.5 ± 8.4; males: n = 236, age: 57.9 ± 8.3.A p value less than 0.05 was considered to be statistically significant.

Table S4. Comparison of serum TFAs levels between four different age groups for HCs

Dependent variable	Age groups		Mean difference	Std. error	*p* value	95% Confidence interval	
						Lower bound	Upper bound
$C_{16:0}$	1	2	-.0464315	.1229590	.706	-.288032	.195169
		3	-.0587764	.1264696	.642	-.307275	.189722
		4	-.1336419	.1468347	.363	-.422156	.154872
	2	1	.0464315	.1229590	.706	-.195169	.288032
		3	-.0123449	.1166002	.916	-.241451	.216762
		4	-.0872104	.1384250	.529	-.359200	.184779
	3	1	.0587764	.1264696	.642	-.189722	.307275
		2	.0123449	.1166002	.916	-.216762	.241451
		4	-.0748655	.1415526	.597	-.353000	.203269
	4	1	.1336419	.1468347	.363	-.154872	.422156
		2	.0872104	.1384250	.529	-.184779	.359200
		3	.0748655	.1415526	.597	-.203269	.353000
$C_{16:1}$	1	2	-.0138501	.1230348	.910	-.255600	.227899
		3	-.0243956	.1265476	.847	-.273047	.224256
		4	-.0697429	.1469252	.635	-.358434	.218949
	2	1	.0138501	.1230348	.910	-.227899	.255600
		3	-.0105455	.1166721	.928	-.239793	.218702
		4	-.0558927	.1385104	.687	-.328050	.216265
	3	1	.0243956	.1265476	.847	-.224256	.273047
		2	.0105455	.1166721	.928	-.218702	.239793
		4	-.0453472	.1416399	.749	-.323654	.232959
	4	1	.0697429	.1469252	.635	-.218949	.358434
		2	.0558927	.1385104	.687	-.216265	.328050
		3	.0453472	.1416399	.749	-.232959	.323654
$C_{18:0}$	1	2	-.0556463	.1228888	.651	-.297109	.185816
		3	-.1333888	.1263974	.292	-.381745	.114968
		4	-.1280558	.1467509	.383	-.416405	.160293
	2	1	.0556463	.1228888	.651	-.185816	.297109
		3	-.0777425	.1165337	.505	-.306718	.151233
		4	-.0724095	.1383460	.601	-.344244	.199425
	3	1	.1333888	.1263974	.292	-.114968	.381745
		2	.0777425	.1165337	.505	-.151233	.306718
		4	.0053331	.1414718	.970	-.272643	.283309

	4	1	.1280558	.1467509	.383	-.160293	.416405
		2	.0724095	.1383460	.601	-.199425	.344244
		3	-.0053331	.1414718	.970	-.283309	.272643
$C_{18:1}$	1	2	-.1059955	.1225979	.388	-.346887	.134895
		3	-.2371665	.1260982	.061	-.484935	.010602
		4	-.1599173	.1464035	.275	-.447584	.127749
	2	1	.1059955	.1225979	.388	-.134895	.346887
		3	-.1311709	.1162578	.260	-.359604	.097263
		4	-.0539218	.1380185	.696	-.325113	.217269
	3	1	.2371665	.1260982	.061	-.010602	.484935
		2	.1311709	.1162578	.260	-.097263	.359604
		4	.0772492	.1411369	.584	-.200069	.354567
	4	1	.1599173	.1464035	.275	-.127749	.447584
		2	.0539218	.1380185	.696	-.217269	.325113
		3	-.0772492	.1411369	.584	-.354567	.200069
$C_{18:2}$	1	2	-.1413415	.1225266	.249	-.382093	.099409
		3	-.2600810*	.1260249	**.040**	-.507706	-.012456
		4	-.1472032	.1463184	.315	-.434702	.140296
	2	1	.1413415	.1225266	.249	-.099409	.382093
		3	-.1187395	.1161902	.307	-.347040	.109561
		4	-.0058617	.1379383	.966	-.276895	.265172
	3	1	.2600810*	.1260249	**.040**	.012456	.507706
		2	.1187395	.1161902	.307	-.109561	.347040
		4	.1128778	.1410548	.424	-.164279	.390035
	4	1	.1472032	.1463184	.315	-.140296	.434702
		2	.0058617	.1379383	.966	-.265172	.276895
		3	-.1128778	.1410548	.424	-.390035	.164279
$C_{18:3}$	1	2	-.1365541	.1228228	.267	-.377887	.104779
		3	-.1437623	.1263295	.256	-.391986	.104461
		4	-.0340796	.1466721	.816	-.322274	.254115
	2	1	.1365541	.1228228	.267	-.104779	.377887
		3	-.0072082	.1164711	.951	-.236061	.221644
		4	.1024746	.1382717	.459	-.169214	.374163
	3	1	.1437623	.1263295	.256	-.104461	.391986
		2	.0072082	.1164711	.951	-.221644	.236061
		4	.1096828	.1413958	.438	-.168144	.387510

		1	.0340796	.1466721	.816	-.254115	.322274
	4	2	-.1024746	.1382717	.459	-.374163	.169214
		3	-.1096828	.1413958	.438	-.387510	.168144
C20:2	1	2	-.2043527	.1203672	.090	-.440861	.032155
		3	-.4243059*	.1238038	**.001**	-.667567	-.181045
		4	-.6052356*	.1437397	**.000**	-.887668	-.322803
	2	1	.2043527	.1203672	.090	-.032155	.440861
		3	-.2199532	.1141425	.055	-.444230	.004324
		4	-.4008828*	.1355073	**.003**	-.667139	-.134626
	3	1	.4243059*	.1238038	**.001**	.181045	.667567
		2	.2199532	.1141425	.055	-.004324	.444230
		4	-.1809297	.1385689	.192	-.453202	.091343
	4	1	.6052356*	.1437397	**.000**	.322803	.887668
		2	.4008828*	.1355073	**.003**	.134626	.667139
		3	.1809297	.1385689	.192	-.091343	.453202
C20:3	1	2	-.1986515	.1209417	.101	-.436288	.038985
		3	-.3573885*	.1243947	**.004**	-.601810	-.112967
		4	-.5583096*	.1444257	**.000**	-.842090	-.274529
	2	1	.1986515	.1209417	.101	-.038985	.436288
		3	-.1587369	.1146873	.167	-.384085	.066611
		4	-.3596581*	.1361540	**.009**	-.627185	-.092131
	3	1	.3573885*	.1243947	**.004**	.112967	.601810
		2	.1587369	.1146873	.167	-.066611	.384085
		4	-.2009212	.1392303	.150	-.474493	.072651
	4	1	.5583096*	.1444257	**.000**	.274529	.842090
		2	.3596581*	.1361540	**.009**	.092131	.627185
		3	.2009212	.1392303	.150	-.072651	.474493
C20:4	1	2	-.1101016	.1214074	.365	-.348654	.128450
		3	-.3105464*	.1248737	**.013**	-.555909	-.065184
		4	-.4636174*	.1449819	**.001**	-.748490	-.178744
	2	1	.1101016	.1214074	.365	-.128450	.348654
		3	-.2004449	.1151289	.082	-.426660	.025771
		4	-.3535158*	.1366783	**.010**	-.622073	-.084958
	3	1	.3105464*	.1248737	**.013**	.065184	.555909
		2	.2004449	.1151289	.082	-.025771	.426660
		4	-.1530709	.1397664	.274	-.427696	.121554

	4	1	.4636174*	.1449819	**.001**	.178744	.748490
		2	.3535158*	.1366783	**.010**	.084958	.622073
		3	.1530709	.1397664	.274	-.121554	.427696
$C_{20:5}$	1	2	-.1795785	.1209465	.138	-.417225	.058068
		3	-.4292703*	.1243996	**.001**	-.673702	-.184839
		4	-.4814897*	.1444314	**.001**	-.765281	-.197698
	2	1	.1795785	.1209465	.138	-.058068	.417225
		3	-.2496918*	.1146918	**.030**	-.475048	-.024335
		4	-.3019112*	.1361594	**.027**	-.569449	-.034373
	3	1	.4292703*	.1243996	**.001**	.184839	.673702
		2	.2496918*	.1146918	**.030**	.024335	.475048
		4	-.0522194	.1392358	.708	-.325802	.221363
	4	1	.4814897*	.1444314	**.001**	.197698	.765281
		2	.3019112*	.1361594	**.027**	.034373	.569449
		3	.0522194	.1392358	.708	-.221363	.325802
$C_{22:5}$	1	2	-.1715503	.1215201	.159	-.410324	.067223
		3	-.3313840*	.1249897	**.008**	-.576975	-.085793
		4	-.4592680*	.1451165	**.002**	-.744406	-.174130
	2	1	.1715503	.1215201	.159	-.067223	.410324
		3	-.1598338	.1152358	.166	-.386259	.066592
		4	-.2877177*	.1368052	**.036**	-.556525	-.018911
	3	1	.3313840*	.1249897	**.008**	.085793	.576975
		2	.1598338	.1152358	.166	-.066592	.386259
		4	-.1278840	.1398962	.361	-.402764	.146996
	4	1	.4592680*	.1451165	**.002**	.174130	.744406
		2	.2877177*	.1368052	**.036**	.018911	.556525
		3	.1278840	.1398962	.361	-.146996	.402764
$C_{22:6}$	1	2	-.1788205	.1212892	.141	-.417140	.059499
		3	-.3917059*	.1247521	**.002**	-.636830	-.146582
		4	-.4524169*	.1448407	**.002**	-.737013	-.167821
	2	1	.1788205	.1212892	.141	-.059499	.417140
		3	-.2128854	.1150168	.065	-.438881	.013110
		4	-.2735964*	.1365452	**.046**	-.541892	-.005300
	3	1	.3917059*	.1247521	**.002**	.146582	.636830
		2	.2128854	.1150168	.065	-.013110	.438881
		4	-.0607110	.1396303	.664	-.335069	.213647

	1	.4524169*	.1448407	**.002**	.167821	.737013
4	2	.2735964*	.1365452	**.046**	.005300	.541892
	3	.0607110	.1396303	.664	-.213647	.335069

HCs were divided into four different age groups: group 1, 30−39 years (*n* = 114); group 2, 40−49 years (*n* = 157); group 3, 50−59 years (*n* = 138), and group 4, 60−70 years (*n* = 78). Data, which are not normal distribution, were transformed to normal distribution before statistic analysis. Continuous variables were analyzed using one-way ANOVA with LSD test. A *p* value less than 0.05 was considered to be statistically significant.

Table S5. Comparison of serum TFAs levels between four different age groups for BLD patients

Dependent variable	Age groups		Mean difference	Std. error	*p* value	95% Confidence interval	
						Lower bound	Upper bound
$C_{16:0}$	1	2	.3282649	.2085139	.116	-.081459	.737989
		3	.1950396	.1916624	.309	-.181571	.571651
		4	.3488491	.1896582	.066	-.023824	.721522
	2	1	-.3282649	.2085139	.116	-.737989	.081459
		3	-.1332253	.1353694	.326	-.399222	.132772
		4	.0205842	.1325165	.877	-.239807	.280975
	3	1	-.1950396	.1916624	.309	-.571651	.181571
		2	.1332253	.1353694	.326	-.132772	.399222
		4	.1538095	.1040051	.140	-.050557	.358177
	4	1	-.3488491	.1896582	.066	-.721522	.023824
		2	-.0205842	.1325165	.877	-.280975	.239807
		3	-.1538095	.1040051	.140	-.358177	.050557
$C_{16:1}$	1	2	.2851419	.2084486	.172	-.124453	.694737
		3	.1618479	.1916024	.399	-.214645	.538341
		4	.3418663	.1895988	.072	-.030690	.714422
	2	1	-.2851419	.2084486	.172	-.694737	.124453
		3	-.1232940	.1353270	.363	-.389208	.142620
		4	.0567243	.1324750	.669	-.203585	.317034
	3	1	-.1618479	.1916024	.399	-.538341	.214645
		2	.1232940	.1353270	.363	-.142620	.389208
		4	.1800183	.1039726	.084	-.024285	.384321
	4	1	-.3418663	.1895988	.072	-.714422	.030690
		2	-.0567243	.1324750	.669	-.317034	.203585
		3	-.1800183	.1039726	.084	-.384321	.024285
$C_{18:0}$	1	2	.3051606	.2087803	.145	-.105087	.715408
		3	.0780072	.1919073	.685	-.299085	.455099
		4	.1691574	.1899006	.374	-.203992	.542306
	2	1	-.3051606	.2087803	.145	-.715408	.105087
		3	-.2271534	.1355423	.094	-.493490	.039183
		4	-.1360032	.1326858	.306	-.396727	.124721
	3	1	-.0780072	.1919073	.685	-.455099	.299085
		2	.2271534	.1355423	.094	-.039183	.493490

		4	.0911502	.1041380	.382	-.113478	.295778
	4	1	-.1691574	.1899006	.374	-.542306	.203992
		2	.1360032	.1326858	.306	-.124721	.396727
		3	-.0911502	.1041380	.382	-.295778	.113478
$C_{18:1}$	1	2	.3530162	.2084341	.091	-.056551	.762583
		3	.1038354	.1915891	.588	-.272632	.480302
		4	.2468563	.1895857	.194	-.125674	.619387
	2	1	-.3530162	.2084341	.091	-.762583	.056551
		3	-.2491808	.1353176	.066	-.515076	.016714
		4	-.1061599	.1324658	.423	-.366451	.154132
	3	1	-.1038354	.1915891	.588	-.480302	.272632
		2	.2491808	.1353176	.066	-.016714	.515076
		4	.1430209	.1039654	.170	-.061268	.347310
	4	1	-.2468563	.1895857	.194	-.619387	.125674
		2	.1061599	.1324658	.423	-.154132	.366451
		3	-.1430209	.1039654	.170	-.347310	.061268
$C_{18:2}$	1	2	.2432998	.2084845	.244	-.166366	.652966
		3	.0303750	.1916354	.874	-.346183	.406933
		4	.2241341	.1896315	.238	-.148486	.596754
	2	1	-.2432998	.2084845	.244	-.652966	.166366
		3	-.2129248	.1353503	.116	-.478884	.053035
		4	-.0191658	.1324978	.885	-.279520	.241189
	3	1	-.0303750	.1916354	.874	-.406933	.346183
		2	.2129248	.1353503	.116	-.053035	.478884
		4	.1937591	.1039905	.063	-.010579	.398097
	4	1	-.2241341	.1896315	.238	-.596754	.148486
		2	.0191658	.1324978	.885	-.241189	.279520
		3	-.1937591	.1039905	.063	-.398097	.010579
$C_{18:3}$	1	2	.2483216	.2082501	.234	-.160884	.657527
		3	.0622448	.1914199	.745	-.313890	.438379
		4	.2856070	.1894182	.132	-.086594	.657808
	2	1	-.2483216	.2082501	.234	-.657527	.160884
		3	-.1860769	.1351981	.169	-.451737	.079583
		4	.0372853	.1323488	.778	-.222776	.297347
	3	1	-.0622448	.1914199	.745	-.438379	.313890
		2	.1860769	.1351981	.169	-.079583	.451737

		4	.2233622*	.1038735	**.032**	.019254	.427471
	4	1	-.2856070	.1894182	.132	-.657808	.086594
		2	-.0372853	.1323488	.778	-.297347	.222776
		3	-.2233622*	.1038735	**.032**	-.427471	-.019254
$C_{20:2}$	1	2	.0223699	.2090396	.915	-.388387	.433127
		3	-.1728052	.1921456	.369	-.550366	.204755
		4	-.1013559	.1901364	.594	-.474968	.272257
	2	1	-.0223699	.2090396	.915	-.433127	.388387
		3	-.1951752	.1357107	.151	-.461843	.071492
		4	-.1237258	.1328506	.352	-.384773	.137322
	3	1	.1728052	.1921456	.369	-.204755	.550366
		2	.1951752	.1357107	.151	-.071492	.461843
		4	.0714494	.1042673	.494	-.133433	.276332
	4	1	.1013559	.1901364	.594	-.272257	.474968
		2	.1237258	.1328506	.352	-.137322	.384773
		3	-.0714494	.1042673	.494	-.276332	.133433
$C_{20:3}$	1	2	.0231715	.2094847	.912	-.388460	.434803
		3	-.0588353	.1925548	.760	-.437200	.319529
		4	-.0277948	.1905412	.884	-.402203	.346613
	2	1	-.0231715	.2094847	.912	-.434803	.388460
		3	-.0820068	.1359996	.547	-.349242	.185229
		4	-.0509663	.1331335	.702	-.312570	.210637
	3	1	.0588353	.1925548	.760	-.319529	.437200
		2	.0820068	.1359996	.547	-.185229	.349242
		4	.0310405	.1044894	.767	-.174278	.236359
	4	1	.0277948	.1905412	.884	-.346613	.402203
		2	.0509663	.1331335	.702	-.210637	.312570
		3	-.0310405	.1044894	.767	-.236359	.174278
$C_{20:4}$	1	2	.2137684	.2087599	.306	-.196439	.623975
		3	-.0425960	.1918886	.824	-.419651	.334459
		4	.0077026	.1898820	.968	-.365410	.380815
	2	1	-.2137684	.2087599	.306	-.623975	.196439
		3	-.2563644	.1355291	.059	-.522675	.009946
		4	-.2060658	.1326728	.121	-.466764	.054632
	3	1	.0425960	.1918886	.824	-.334459	.419651
		2	.2563644	.1355291	.059	-.009946	.522675

		4	.0502986	.1041278	.629	-.154310	.254907
	4	1	-.0077026	.1898820	.968	-.380815	.365410
		2	.2060658	.1326728	.121	-.054632	.466764
		3	-.0502986	.1041278	.629	-.254907	.154310
$C_{20:5}$	1	2	.1861875	.2087866	.373	-.224072	.596447
		3	-.0495615	.1919131	.796	-.426665	.327542
		4	-.0456612	.1899062	.810	-.418821	.327499
	2	1	-.1861875	.2087866	.373	-.596447	.224072
		3	-.2357490	.1355464	.083	-.502094	.030596
		4	-.2318487	.1326898	.081	-.492580	.028883
	3	1	.0495615	.1919131	.796	-.327542	.426665
		2	.2357490	.1355464	.083	-.030596	.502094
		4	.0039003	.1041411	.970	-.200734	.208535
	4	1	.0456612	.1899062	.810	-.327499	.418821
		2	.2318487	.1326898	.081	-.028883	.492580
		3	-.0039003	.1041411	.970	-.208535	.200734
$C_{22:5}$	1	2	.1050152	.2089383	.615	-.305543	.515573
		3	-.1189838	.1920526	.536	-.496361	.258394
		4	-.0148248	.1900443	.938	-.388256	.358607
	2	1	-.1050152	.2089383	.615	-.515573	.305543
		3	-.2239990	.1356449	.099	-.490537	.042539
		4	-.1198400	.1327862	.367	-.380761	.141081
	3	1	.1189838	.1920526	.536	-.258394	.496361
		2	.2239990	.1356449	.099	-.042539	.490537
		4	.1041590	.1042168	.318	-.100624	.308942
	4	1	.0148248	.1900443	.938	-.358607	.388256
		2	.1198400	.1327862	.367	-.141081	.380761
		3	-.1041590	.1042168	.318	-.308942	.100624
$C_{22:6}$	1	2	.2967949	.2087149	.156	-.113324	.706914
		3	.0515226	.1918472	.788	-.325452	.428497
		4	.0779597	.1898411	.682	-.295072	.450992
	2	1	-.2967949	.2087149	.156	-.706914	.113324
		3	-.2452723	.1354999	.071	-.511526	.020981
		4	-.2188352	.1326443	.100	-.479477	.041807
	3	1	-.0515226	.1918472	.788	-.428497	.325452
		2	.2452723	.1354999	.071	-.020981	.511526

	4	.0264371	.1041054	.800	-.178127	.231001
4	1	-.0779597	.1898411	.682	-.450992	.295072
	2	.2188352	.1326443	.100	-.041807	.479477
	3	-.0264371	.1041054	.800	-.231001	.178127

BLD patients were divided into four different age groups: group 1, 30-39 years (*n* = 32); group 2, 40-49 years (*n* = 79); group 3, 50-59 years (*n* = 171), and group 4, 60-70 years (*n* = 197). Data, which are not normal distribution, were transformed to normal distribution before statistic analysis. Continuous variables were analyzed using one-way ANOVA with LSD test. A *p* value less than 0.05 was considered to be statistically significant.

Table S6. Comparison of serum TFAs levels between four different age groups for LC patients

Dependent variable	Age groups		Mean difference	Std. error	*p* value	95% Confidence interval	
						Lower bound	Upper bound
$C_{16:0}$	1	2	-.0642324	.2859335	.822	-.626099	.497634
		3	-.2834261	.2695256	.294	-.813050	.246198
		4	-.1523706	.2652808	.566	-.673654	.368913
	2	1	.0642324	.2859335	.822	-.497634	.626099
		3	-.2191937	.1480994	.140	-.510213	.071825
		4	-.0881383	.1402259	.530	-.363686	.187409
	3	1	.2834261	.2695256	.294	-.246198	.813050
		2	.2191937	.1480994	.140	-.071825	.510213
		4	.1310554	.1027103	.203	-.070773	.332884
	4	1	.1523706	.2652808	.566	-.368913	.673654
		2	.0881383	.1402259	.530	-.187409	.363686
		3	-.1310554	.1027103	.203	-.332884	.070773
$C_{16:1}$	1	2	-.3606735	.2855540	.207	-.921794	.200447
		3	-.5090541	.2691679	.059	-1.037976	.019867
		4	-.3791529	.2649288	.153	-.899744	.141439
	2	1	.3606735	.2855540	.207	-.200447	.921794
		3	-.1483806	.1479028	.316	-.439013	.142252
		4	-.0184794	.1400398	.895	-.293661	.256702
	3	1	.5090541	.2691679	.059	-.019867	1.037976
		2	.1483806	.1479028	.316	-.142252	.439013
		4	.1299011	.1025740	.206	-.071659	.331462
	4	1	.3791529	.2649288	.153	-.141439	.899744
		2	.0184794	.1400398	.895	-.256702	.293661
		3	-.1299011	.1025740	.206	-.331462	.071659
$C_{18:0}$	1	2	.1290363	.2864224	.653	-.433791	.691863
		3	-.0111873	.2699865	.967	-.541717	.519343
		4	.1037384	.2657344	.696	-.418436	.625913
	2	1	-.1290363	.2864224	.653	-.691863	.433791
		3	-.1402236	.1483526	.345	-.431740	.151293
		4	-.0252979	.1404657	.857	-.301316	.250721
	3	1	.0111873	.2699865	.967	-.519343	.541717
		2	.1402236	.1483526	.345	-.151293	.431740

		4	.1149257	.1028860	.265	-.087248	.317099
	4	1	-.1037384	.2657344	.696	-.625913	.418436
		2	.0252979	.1404657	.857	-.250721	.301316
		3	-.1149257	.1028860	.265	-.317099	.087248
$C_{18:1}$	1	2	-.2347542	.2858494	.412	-.796455	.326947
		3	-.3981753	.2694464	.140	-.927644	.131293
		4	-.2613999	.2652029	.325	-.782530	.259730
	2	1	.2347542	.2858494	.412	-.326947	.796455
		3	-.1634211	.1480559	.270	-.454354	.127512
		4	-.0266457	.1401847	.849	-.302112	.248821
	3	1	.3981753	.2694464	.140	-.131293	.927644
		2	.1634211	.1480559	.270	-.127512	.454354
		4	.1367754	.1026801	.183	-.064994	.338544
	4	1	.2613999	.2652029	.325	-.259730	.782530
		2	.0266457	.1401847	.849	-.248821	.302112
		3	-.1367754	.1026801	.183	-.338544	.064994
$C_{18:2}$	1	2	.1527553	.2867389	.594	-.410694	.716204
		3	.1163021	.2702848	.667	-.414814	.647418
		4	.0716968	.2660281	.788	-.451055	.594448
	2	1	-.1527553	.2867389	.594	-.716204	.410694
		3	-.0364532	.1485166	.806	-.328292	.255385
		4	-.0810585	.1406209	.565	-.357382	.195265
	3	1	-.1163021	.2702848	.667	-.647418	.414814
		2	.0364532	.1485166	.806	-.255385	.328292
		4	-.0446052	.1029997	.665	-.247002	.157792
	4	1	-.0716968	.2660281	.788	-.594448	.451055
		2	.0810585	.1406209	.565	-.195265	.357382
		3	.0446052	.1029997	.665	-.157792	.247002
$C_{18:3}$	1	2	-.3133304	.2861817	.274	-.875684	.249024
		3	-.3817233	.2697596	.158	-.911807	.148361
		4	-.2862577	.2655111	.282	-.807993	.235478
	2	1	.3133304	.2861817	.274	-.249024	.875684
		3	-.0683930	.1482280	.645	-.359665	.222879
		4	.0270727	.1403476	.847	-.248714	.302859
	3	1	.3817233	.2697596	.158	-.148361	.911807
		2	.0683930	.1482280	.645	-.222879	.359665

		4	.0954657	.1027995	.354	-.106538	.297469
	4	1	.2862577	.2655111	.282	-.235478	.807993
		2	-.0270727	.1403476	.847	-.302859	.248714
		3	-.0954657	.1027995	.354	-.297469	.106538
$C_{20:2}$	1	2	-.0911460	.2862102	.750	-.653556	.471264
		3	-.1495683	.2697865	.580	-.679705	.380569
		4	.0019173	.2655376	.994	-.519870	.523705
	2	1	.0911460	.2862102	.750	-.471264	.653556
		3	-.0584223	.1482427	.694	-.349723	.232878
		4	.0930634	.1403616	.508	-.182751	.368877
	3	1	.1495683	.2697865	.580	-.380569	.679705
		2	.0584223	.1482427	.694	-.232878	.349723
		4	.1514857	.1028097	.141	-.050538	.353509
	4	1	-.0019173	.2655376	.994	-.523705	.519870
		2	-.0930634	.1403616	.508	-.368877	.182751
		3	-.1514857	.1028097	.141	-.353509	.050538
$C_{20:3}$	1	2	-.1404536	.2864463	.624	-.703327	.422420
		3	-.2245290	.2700090	.406	-.755103	.306045
		4	-.1144553	.2657566	.667	-.636674	.407763
	2	1	.1404536	.2864463	.624	-.422420	.703327
		3	-.0840754	.1483650	.571	-.375616	.207465
		4	.0259982	.1404774	.853	-.250043	.302040
	3	1	.2245290	.2700090	.406	-.306045	.755103
		2	.0840754	.1483650	.571	-.207465	.375616
		4	.1100737	.1028945	.285	-.092117	.312264
	4	1	.1144553	.2657566	.667	-.407763	.636674
		2	-.0259982	.1404774	.853	-.302040	.250043
		3	-.1100737	.1028945	.285	-.312264	.092117
$C_{20:4}$	1	2	.2275207	.2858603	.426	-.334202	.789243
		3	.0025893	.2694567	.992	-.526900	.532078
		4	.1587742	.2652130	.550	-.362376	.679924
	2	1	-.2275207	.2858603	.426	-.789243	.334202
		3	-.2249314	.1480615	.129	-.515876	.066013
		4	-.0687465	.1401900	.624	-.344223	.206730
	3	1	-.0025893	.2694567	.992	-.532078	.526900
		2	.2249314	.1480615	.129	-.066013	.515876

		4	.1561849	.1026841	.129	-.045592	.357962
	4	1	-.1587742	.2652130	.550	-.679924	.362376
		2	.0687465	.1401900	.624	-.206730	.344223
		3	-.1561849	.1026841	.129	-.357962	.045592
$C_{20:5}$	1	2	.0081655	.2863941	.977	-.554606	.570937
		3	-.1228659	.2699598	.649	-.653343	.407612
		4	.0030193	.2657082	.991	-.519104	.525142
	2	1	-.0081655	.2863941	.977	-.570937	.554606
		3	-.1310314	.1483380	.378	-.422519	.160456
		4	-.0051463	.1404518	.971	-.281137	.270845
	3	1	.1228659	.2699598	.649	-.407612	.653343
		2	.1310314	.1483380	.378	-.160456	.422519
		4	.1258852	.1028758	.222	-.076268	.328039
	4	1	-.0030193	.2657082	.991	-.525142	.519104
		2	.0051463	.1404518	.971	-.270845	.281137
		3	-.1258852	.1028758	.222	-.328039	.076268
$C_{22:5}$	1	2	.0738344	.2858018	.796	-.487773	.635442
		3	-.1010230	.2694015	.708	-.630403	.428357
		4	.0893534	.2651587	.736	-.431690	.610397
	2	1	-.0738344	.2858018	.796	-.635442	.487773
		3	-.1748574	.1480312	.238	-.465742	.116027
		4	.0155190	.1401613	.912	-.259901	.290939
	3	1	.1010230	.2694015	.708	-.428357	.630403
		2	.1748574	.1480312	.238	-.116027	.465742
		4	.1903764	.1026630	.064	-.011359	.392112
	4	1	-.0893534	.2651587	.736	-.610397	.431690
		2	-.0155190	.1401613	.912	-.290939	.259901
		3	-.1903764	.1026630	.064	-.392112	.011359
$C_{22:6}$	1	2	.1063574	.2859990	.710	-.455637	.668352
		3	-.1304968	.2695873	.629	-.660242	.399249
		4	-.0010075	.2653416	.997	-.522410	.520395
	2	1	-.1063574	.2859990	.710	-.668352	.455637
		3	-.2368542	.1481333	.111	-.527940	.054231
		4	-.1073649	.1402580	.444	-.382975	.168245
	3	1	.1304968	.2695873	.629	-.399249	.660242
		2	.2368542	.1481333	.111	-.054231	.527940

	4	.1294893	.1027339	.208	-.072385	.331364
	1	.0010075	.2653416	.997	-.520395	.522410
4	2	.1073649	.1402580	.444	-.168245	.382975
	3	-.1294893	.1027339	.208	-.331364	.072385

LC patients were divided into four different age groups: group 1, 30−39 years (n = 15); group 2, 40−49 years (n = 64); group 3, 50−59 years (n = 155), and group 4, 60−70 years (n = 240). Data, which are not normal distribution, were transformed to normal distribution before statistic analysis. Continuous variables were analyzed using one-way ANOVA with LSD test. A p value less than 0.05 was considered to be statistically significant.

Table S7. Comparison of serum TFA levels between three pathophysiological states in the training set and validation set using Mann-Whitney U test

TFAs	Training set			Validation set		
	HC *vs.*BLD	HC *vs.*LC	BLD *vs.*LC	HC *vs.*BLD	HC *vs.*LC	BLD *vs.*LC
$C_{16:0}$	0.834	**0.047**	**0.025**	0.372	**0.030**	**0.024**
$C_{16:1}$	0.753	0.050	**0.031**	0.544	0.054	**0.009**
$C_{18:0}$	0.850	**0.046**	**0.042**	0.662	**3.528E-08**	**4.971E-07**
$C_{18:1}$	0.225	**0.022**	**0.001**	0.671	**2.496E-04**	**5.612E-05**
$C_{18:2}$	**0.046**	0.161	0.585	**0.002**	0.176	0.304
$C_{18:3}$	0.992	**0.034**	**0.034**	0.387	**6.130E-05**	**0.001**
$C_{20:2}$	**1.719E-05**	**0.005**	0.306	**2.037E-09**	**6.067E-09**	0.503
$C_{20:3}$	**3.617E-05**	0.050	**0.028**	**8.906E-07**	0.054	**0.017**
$C_{20:4}$	**2.222E-05**	**0.002**	0.217	**1.312E-15**	**8.105E-10**	0.080
$C_{20:5}$	**0.001**	**0.019**	0.250	**5.751E-09**	**8.988E-08**	0.872
$C_{22:5}$	**1.508E-08**	**2.109E-04**	0.060	**6.609E-15**	**7.641E-08**	0.089
$C_{22:6}$	**3.422E-09**	**0.001**	**0.003**	**1.529E-22**	**3.686E-09**	**1.475E-05**

A *p* value less than 0.05 was considered to be statistically significant.
